# Evaluation of a cross-border field simulation exercise on the response to outbreaks of infectious diseases in Namanga, Kenya and Tanzania

**DOI:** 10.1371/journal.pgph.0003832

**Published:** 2024-10-16

**Authors:** John Sackey Dzaba, Hilary Kagume Njenge, James Wakhungu, Ralf Reintjes, Nicola Watt

**Affiliations:** 1 Department of Health Sciences, Hamburg University of Applied Sciences, Hamburg, Germany; 2 Health Emergencies Programme, World Health Organization, Addis Ababa, Ethiopia; 3 Directorate of Veterinary Services, Ministry of Agriculture, Nairobi, Kenya; 4 Global Programme for Pandemic Prevention and Response, One Health, Deutsche Gesellschaft für Internationale Zusammenarbeit (GmbH), Bonn, Germany; Georgetown University, UNITED STATES OF AMERICA

## Abstract

Travel and trade, whilst playing a critical role in economic development, contribute to the spread of infectious diseases, including novel or emerging diseases, which can threaten health security locally, regionally and globally. The World Health Organization mandates preparedness through field simulation exercises to address infectious disease outbreaks, as highlighted by the COVID-19 pandemic. This study assessed the impact of the 2019 Namanga field simulation exercise, conducted in the border town shared by Kenya and Tanzania, on improving cross-border outbreak preparedness and response. It focused on participants’ knowledge, skills acquisition and real-world application. An anonymous online survey was administered to participants 37 months post-field simulation exercise. In addition, key informant interviews and a focus group discussion with the Joint Border Management Committee in Namanga were conducted. The June 2019 field simulation exercise enhanced the skills, knowledge, and confidence of participants, including members of the border community, in preparing for and responding to outbreaks including COVID-19. The skills and knowledge gained were deemed valuable, relevant, and effective for use in future response activities. The analysis is limited by potential response bias, as only participants with positive experiences of the field simulation exercise may have responded more favourably. Addressing the limitations of design and implementation of the field simulation exercise and the challenges of cross-border response identified in this study are critical to optimising future responses.

## Introduction

Whilst travel and trade play an important role in most economies, their global scale can exacerbate health systems challenges and threaten global health security, which can lead to increases in morbidity, mortality, and economic burdens [1–3]. The World Health Organization’s (WHO) International Health Regulations (IHR) mandate member states to strengthen public health capacities at Points of Entry (PoE) like border crossings [4]. Under the IHR (2005), the IHR Monitoring and Evaluation Framework (MEF) outlines methods for assessing the implementation of key national public health competencies. These methods include the mandatory State Party Self-Assessment Annual Reports (SPARs), and the voluntary Joint External Evaluation (JEE), After-Action Reviews (AARs), and Simulation Exercises (SimExs).

SimEx includes Field Simulation Exercise (FSX), simulates infectious disease outbreaks to assess contingency plans, resource allocation, and inter-agency coordination. These exercises rigorously test capabilities, systems, and procedures by assessing personnel deployment and resource management, ultimately identifying strengths and weaknesses to enhance overall emergency response capacity [5, 6].

The East African region is consistently threatened by recurring outbreaks of emerging and re-emerging infectious diseases. These outbreaks pose a risk to both human and animal health, as well as socio-economic stability [7]. The East African Community (EAC) Secretariat, in collaboration with the German Government’s "Support to Pandemic Preparedness in the EAC Region" programme and following a mandate from the 11^th^ Ordinary Session of the EAC Sectoral Health Council, organized the cross-border FSX. The exercise took place in June 2019 at Namanga, a PoE designated under the IHR for both Kenya and Tanzania. Namanga was selected as the primary FSX site, along with 23 other sites—12 in Kenya and 11 in Tanzania—due to its status as one of the five official border crossings and high tourist traffic from nearby wildlife parks, which increases the risk of rapid international spread of disease. This study focuses on Namanga as the primary FSX site [8, 9].

The FSX was therefore designed to assess countries’ preparedness and response capabilities particularly at the PoE—in the event of a hypothetical transboundary Rift Valley fever outbreak. The exercise addressed Rift Valley Fever scenarios, including surveillance, early warning mechanisms, outbreak detection aligned with the One Health approach for integrated human, animal, and environmental health. It covered case management, communication strategies, cross-border coordination, logistics, response time, and SOPs. Additionally, it acquainted participants with their roles and tested contingency plans. It involved approximately 300 individuals from 10 African countries [8].

Given resource limitations, it is critical to understand whether tools designed to enhance capacities do have the intended effect. This study therefore aimed to document the benefits and challenges of the Namanga 2019 FSX.

A literature review and discussions with organizers of the Namanga FSX was first conducted to synthesise existing literature and identify gaps in knowledge and methods regarding the impact of SimEx on improving responses to real-world outbreaks. Studies indicate that SimEx, including FSX, can enhance preparedness and response capacities. The benefits of SimEx include increased confidence, improved pre-planning knowledge, and enhanced understanding of individual roles [10]. SimEx helped to identify deficiencies in plans, protocols, communicating, training, and resource allocation [11, 12]. An improvement in the surveillance skills of SimEx participants was observed from pre- and postexercise assessment result [13]. A scoping review study indicated that research on SimEx predominantly utilizes surveys, focus groups or qualitative methods. Additionally, the study noted that there is insufficient empirical evidence on the effectiveness of exercises. Consequently, the study recommended conducting further research to assess the impact of SimEx on real-life emergency responses to strengthen the evidence base [14].

The results of this literature review informed the evaluation framework and data collection methods for this study, which aimed to assess how FSX enhanced participants preparedness to public health emergency, skills and knowledge. In addition, the limitations of FSX are explored and corresponding recommendations identified.

## Methods

This study used an explanatory sequential mixed methods design. A post-exercise online survey (S1 Fig) was followed by a focus group discussion (FGD) and key informant interviews (KII) to augment the understanding of the quantitative findings.

### Data collection methods

#### Quantitative data collection

A survey was conducted in June 2019 to gather insights into the structure, design, and implementation of the FSX [8].

In line with the findings of the research literature and the post-exercise report, the content of the 20-item survey was developed [8, 15]. Construct and content validity was ensured through consultations with two pandemic preparedness experts. The anonymous online survey was conducted via Lime Survey between 10 July and 30 August 2022, 37 months after FSX. The self-administered, semi-structured questionnaire took 5–15 minutes to complete. The three-year period between the FSX survey and the follow-up study was chosen to assess the long-term sustainability and impact of the improvements made in order to obtain mature data and meaningful insights into whether the improvements from the FSX led to sustainable results. Participants in the post-FSX survey were purposively selected from stakeholders involved in the exercise, with a minimum target of 50 respondents. Notably, surveys were conducted only in 2019 and post-FSX in 2022.

#### Qualitative data collection.

Qualitative data was collected during a two-week visit to Kenya and Tanzania between September and October of 2022.

#### FGD

A FGD was conducted with the Kenyan and Tanzanian Joint Border Management Committee (JBMC) in Namanga, including their representatives and some members who participated in the FSX. The discussion guide (S3 Fig) was designed to align with the FSX objectives, survey results, and relevant literature. A pilot study with a Kenyan veterinary officer informed the final guide. Nine JBMC members were recruited using purposive sampling with a target of 5–10 individuals. The discussion lasted for 90 minutes.

#### KII

A semi-structured approach, comprising open-ended questions, was employed to elicit in-depth insights for the KII. This format provided flexibility, allowing participants to elaborate on their responses and discuss additional relevant issues, thereby enriching the study’s data [16].

Fourteen individuals who participated in the FSX were purposively selected for the KII, including international experts, government officials and district staff from various organisations. The aim was to explore experiences beyond the JBMC, with a target of at least 5 participants. The interviews lasted an average of 40 minutes and were recorded and transcribed verbatim.

#### Ethical considerations.

The study upheld informed consent, voluntary participation, and confidentiality during data collection, with participants receiving information sheets and signed consent forms, and obtaining ethical approval (protocol number 2022–15) from the Hamburg University of Applied Sciences.

### Data analysis

#### Quantitative analysis of survey data (2019 & 2022).

The data analysis was conducted using SPSS version 27. Thematic and descriptive analysis summarized responses in frequencies and percentages for each year survey, which informed the guides for the KI interviews (S2 Fig) and the FGD. The 2019 survey’s anonymity necessitated an independent analysis of each year’s results to enable direct comparisons between the two surveys.

#### Qualitative analysis.

Themes and patterns were identified in the open-ended survey responses of 2022 through content analysis [17, 18]. Verbatim transcriptions of the KI interviews and the FGD were uploaded and analysed using MAXQDA 2022 [19]. The study employed a deductive-inductive approach to analyse the FGD and KI interview transcripts. The deductive coding aligned with Kirkpatrick’s model, assessed participant reactions, knowledge gain, skill application, preparedness impact, and the research questions [20]. Inductive coding was used to identify new themes, refine codes, categories and subcategories, and triangulation of qualitative and quantitative data was used to identify areas of convergence and divergence [21].

## Results

### Quantitative results

Of the approximately 300 participants and facilitators involved in the 2019 Namanga FSX, 121 completed the initial survey. In 2022, a retrospective anonymous online survey was sent to the 171 participants whose email addresses were still available. Of these, 57 completed the survey, with 17 emails undeliverable, resulting in an overall response rate of 37%. Since the previous survey was also anonymous, there was no opportunity to retrieve the exact comparable 2019 data set for those 57 participants.

Table 1 illustrates the distribution of self-reported skills and knowledge in 2019 and 2022, with responses for each survey presented independently. Most respondents fully agreed in both years that the FSX enabled participants to test their response capacities; 2019 (69.3%) and 2022 (77.6%). Many respondents in 2019 (60.7%) and almost all in 2022 (93.9%) agreed that the exercise had enhanced their understanding of emergency response roles. In both surveys, a majority of participants (78.6% and 68.8%, respectively) fully agreed that the FSX had helped their organizations identify strengths and gaps in their understanding of response systems, plans, and procedures. In both years, almost half of respondents (56.8% and 45.6% respectively) reported that their organizations were better prepared for a public health emergency.

**Table 1:** Distribution of the FSX participants’ self-reported skills and knowledge (2019 & 2022).

Skills and Knowledge	Response option	2019 (n = 121)	2022 (n = 57)
Did the exercise allow you to test your response capacity?	Fully agree	79 (69.3%)	38 (77.6%)
	Partly agree	23 (20.2%)	11 (19.3%)
	Neutral	4 (3.5%)	0 (0%)
	Partly disagree	4 (3.5%)	0 (0%)
	Strongly disagree	4 (3.5%)	0 (0%)
Did the exercise improve your understanding of your role and function during an emergency response?	Fully agree	68 (60.7%)	46 (93.9%)
	Partly agree	24 (21.4%)	3 (6.1%)
	Neutral	15 (13.4%)	0 (0%)
	Partly disagree	5 (4.5%)	0 (0%)
	Strongly disagree	0 (0%)	0 (0%)
Did the exercise help your organization to identify some of their strengths as well as some of the gaps in your understanding of response systems, plans and procedures?	Fully agree	92 (78.6%)	33 (68.8%)
	Partly agree	17 (14.5%)	14 (29.2%)
	Neutral	6 (5.1%)	1 (2.1%)
	Partly disagree	2 (1.7%)	0 (0%)
	Strongly disagree	0 (0%)	0 (0%)
As a result of the exercise, my organization was better prepared for a health emergency.	Fully agree	67 (56.8%)	26 (45.6%)
	Partly agree	35 (29.7%)	19 (33.3%)
	Neutral	12 (10.2%)	3 (5.3%)
	Partly disagree	4 (3.4%)	0 (0%)
	Strongly disagree	0 (0%)	0 (0%)

#### 2022 survey responses

**Fig 1:**
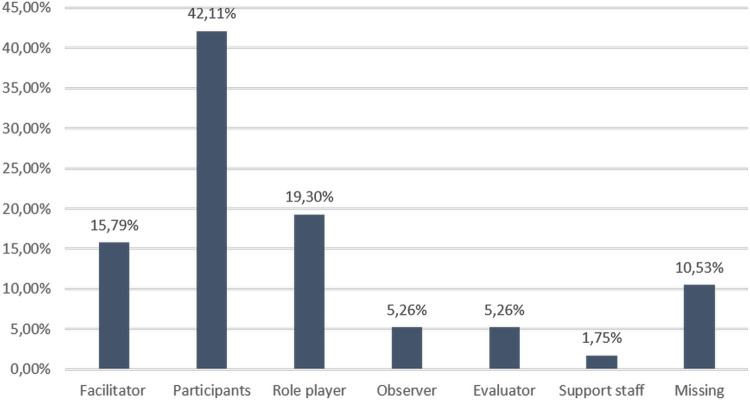
Roles of participants in the 2019 FSX and 2022 survey respondents.

Fig 1 shows that in the 2019 FSX and 2022 survey, respondents included 42,11% (n = 24) and 15,79% (n = 9) facilitators.

**Fig 2:**
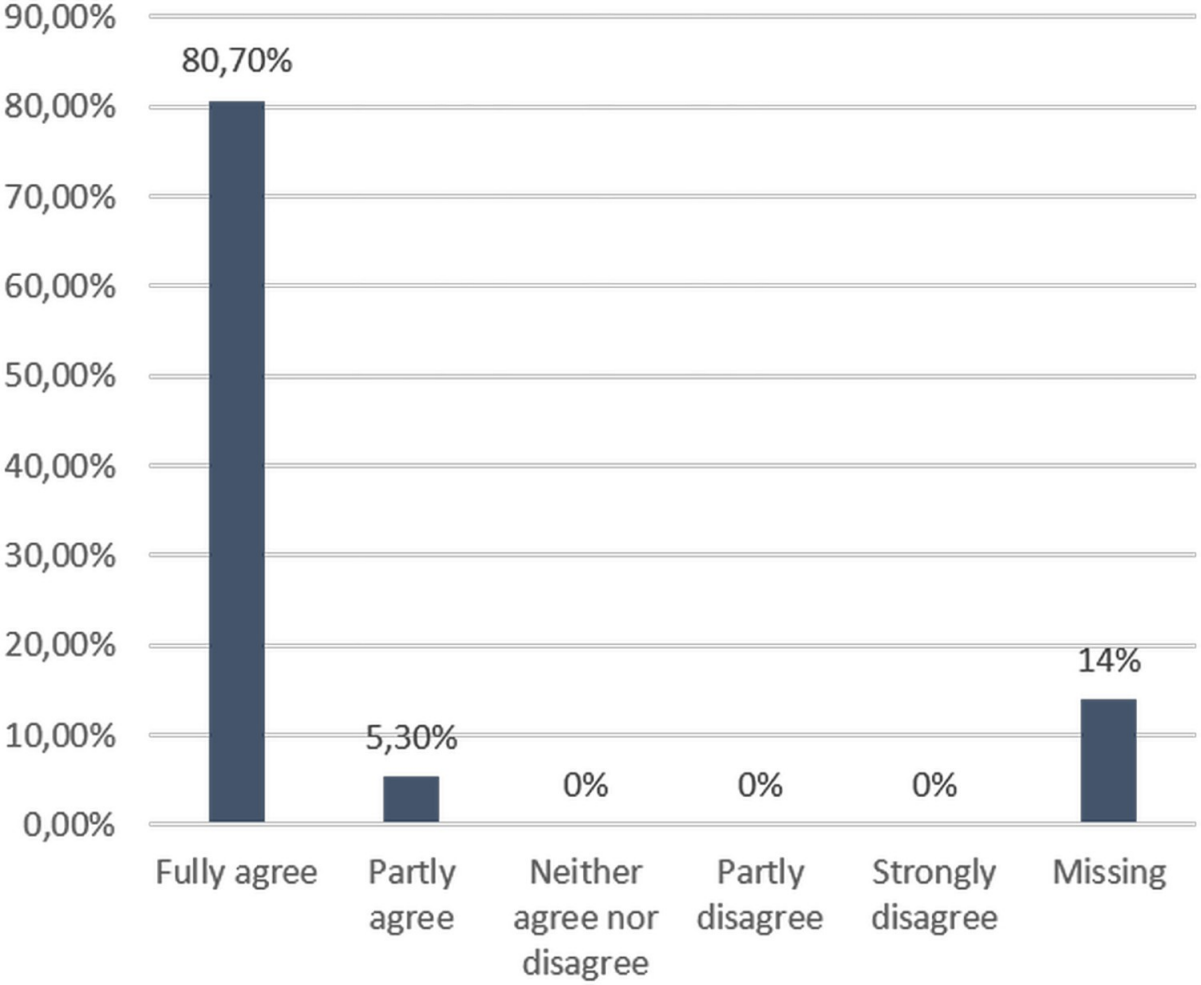
Participants’ responses regarding whether they acquired new skills and knowledge from the FSX.

Fig 2 shows that 80.7% (n = 46) of the respondents agreed that they acquired new skills and knowledge from the FSX, while 5.3% (n = 3) partially agreed with this statement.

**Fig 3:**
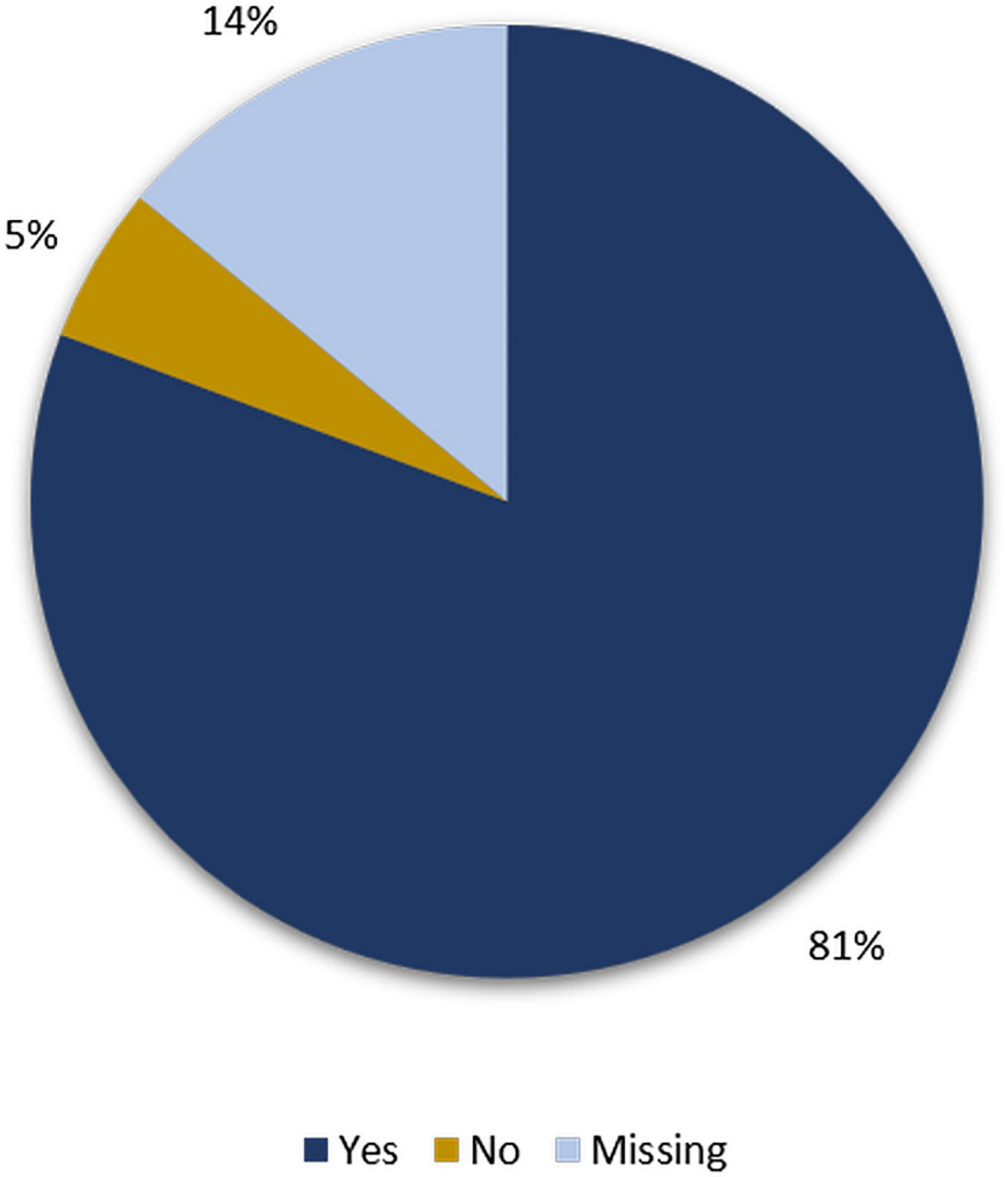
Participants’ responses to the question whether the FSX has improved their understanding of infectious disease outbreaks.

**Fig 4:**
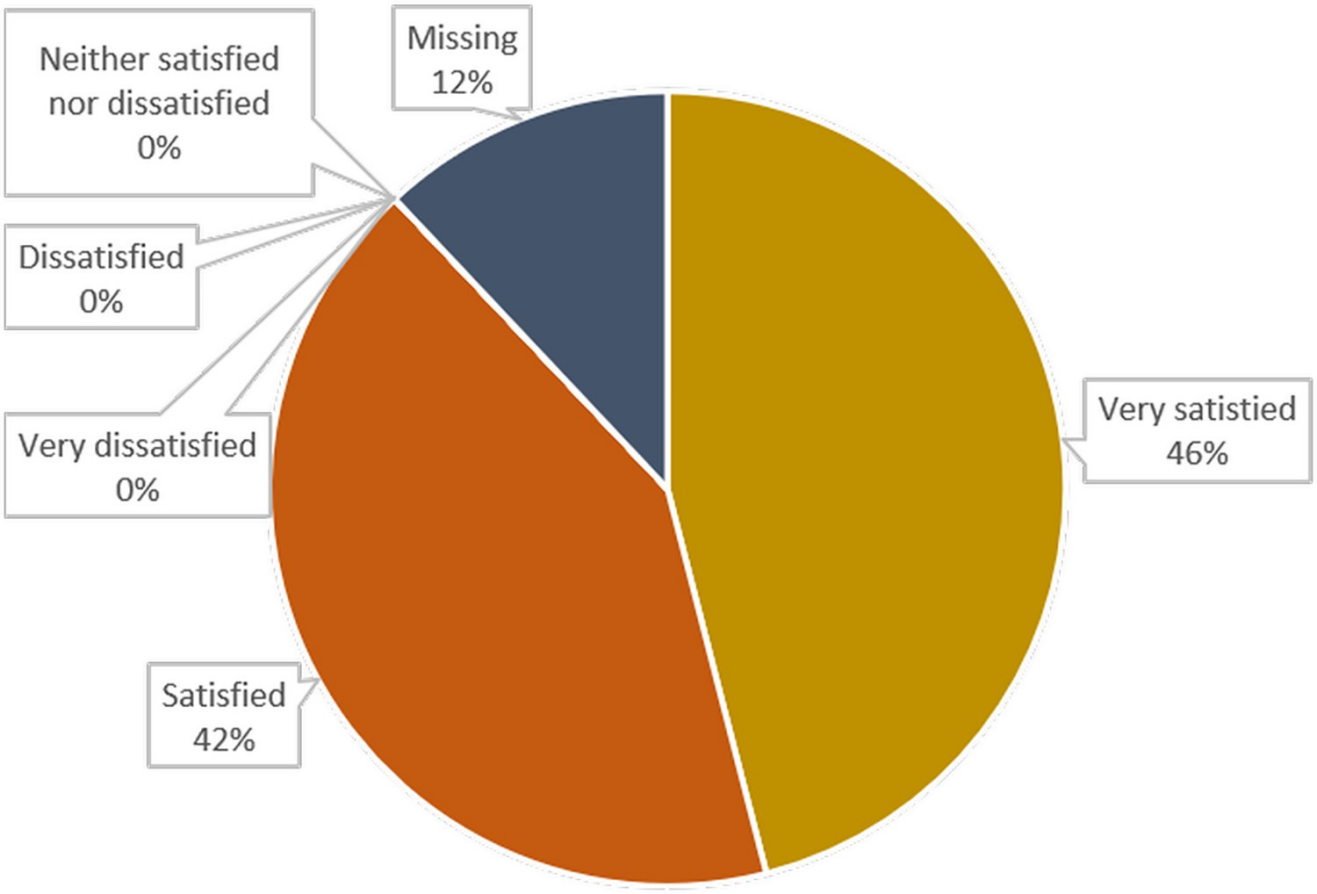
Participants’ overall satisfaction with the FSX.

Fig 3 shows that 81% or n = 46 of the respondents said they had a better understanding and knowledge of infectious disease outbreaks. The majority of respondents expressed satisfaction with the FSX (Fig 4).

**Fig 5:**
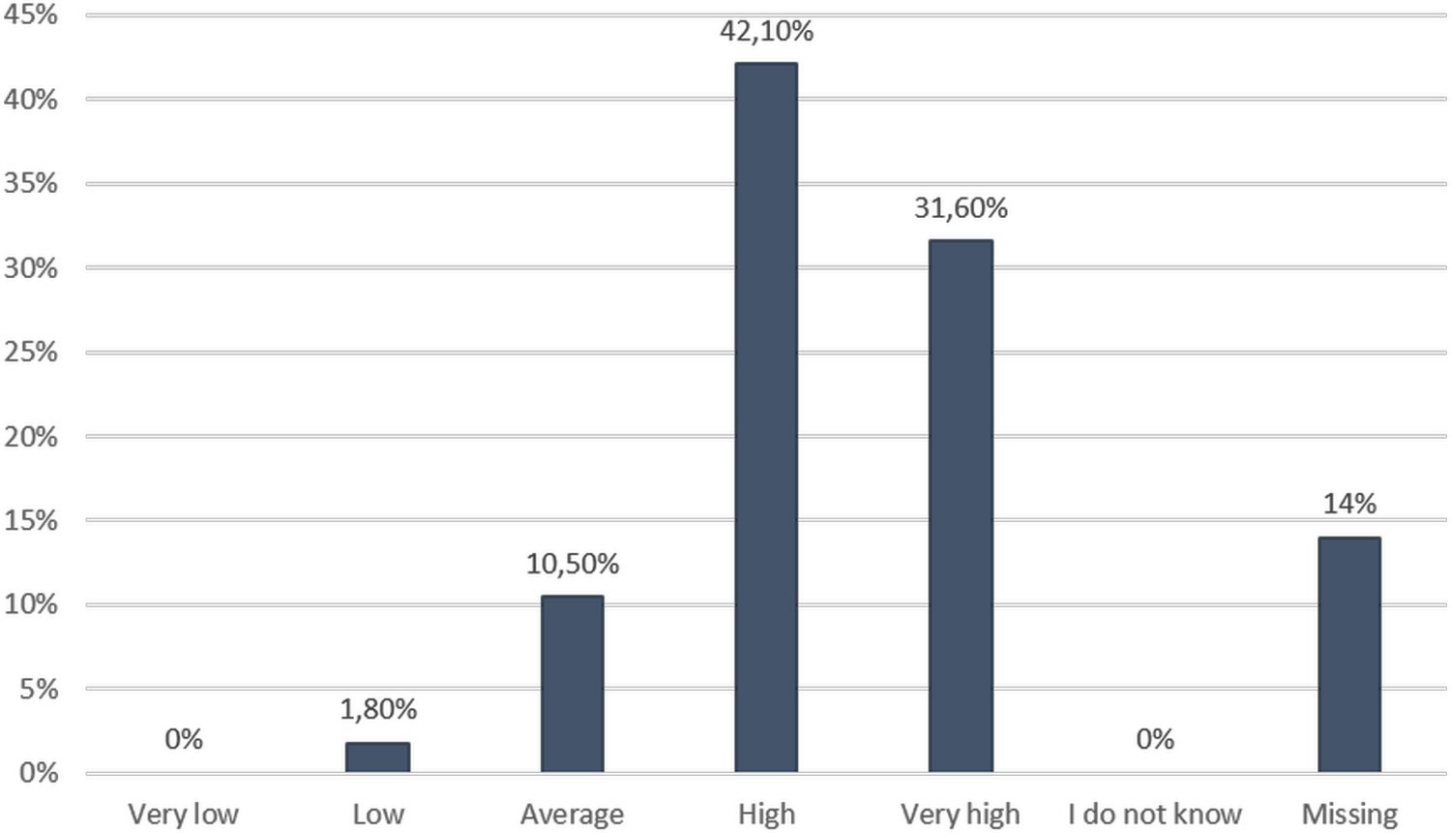
Participants’ responses to how well the skills and knowledge gained in the FSX prepared them for their work in COVID-19 and other infectious diseases.

Nearly three-quarters of the participants reported that the skills and knowledge they had acquired prepared them for their work in responding to COVID-19 and other infectious diseases (Fig 5).

### Qualitative results

The suggestions for improvement captured in the 2022 survey were categorized by theme and the frequency of each theme’s occurrence (Table 2).

**Table 2:** Participants’ feedback on how the FSX could be improved.

Feedback Theme	Summary statistics
Planning and Logistics	12/57 (21.05%) of the participants recommended the inclusive involvement of all FSX participants across sectors, emphasising advanced preparation, improved planning efficiency, blended training methods and improved logistical arrangements.
Pre-training	10/57 (17.54%) of the participants recommended pre-training for the next FSX, including orientations, rehearsals, drills and functional exercises, defining roles prior to the exercise and familiarising themselves with the necessary preparedness tools in their environment.
Resources and time	7/57 (12.28%) of the respondents suggested a more comprehensive next FSX with more practice days and preparation time, along with a budget for post-FSX activities.
Regular FSX	6/57 (10.53%) respondents said that the FSX should be run regularly to remind participants how to prepare for disease outbreaks.
Communication and coordination	5/57 (8.77%) of participants suggested that better communication and coordination during the exercise could be achieved through better network coverage, especially Wi-Fi and mobile phones.

### Thematic analysis.

Reaction (level 1): Participants found the FSX to be useful, relevant, and knowledge-enhancing, increasing confidence, and valued information sharing and collaboration across agencies and countries:

“*The FSX was very useful and relevant to us” (KI-3, Epidemiologist)*

“*We shared a lot with other colleagues from Arusha and Nairobi*, *which helped us to build trust and work together”* (KI-7, Veterinary officer).

To enhance FSX planning and response capability, regular training and highly coordinated structures were recommended by the participants:

“*The exercise should be more comprehensive”* (KI-12, Laboratory Services Coordinator)

*“Currently*, *we all know that there are emerging and re-emerging diseases*, *and for this reason you may not be properly acquainted with the symptoms*, *the science*, *the risk factors […]*. *And for that reason you need to put people on regular training*, *refresher training”* (KI-2, Veterinary officer)

“*Yes*, *we should have very coordinated structures to improve on the FSX”* (KI-2, Veterinary officer).

Participants requested additional training on collaboration, coordination, negotiation, diplomacy, and communication related to infectious diseases and emphasized the importance of training covering a range of infectious diseases:

“*We need more training on collaboration, especially on how to disseminate information and notification of diseases from one level to another […]”* (KI-8, Veterinary officer)

“*I think*, *in the next exercise*, *we could work on several scenarios […]* (KI-1, Defence officer).

This study identified the need for improved border infrastructure, particularly at the Namanga health centre, which lacks quarantine areas, diagnostics and resources to apply the knowledge gained:

“*In terms of diagnostics, we did not have the equipment to help us. Even our region in the Longido district, we were alerted that there is Ebola in Uganda. But we do not have any diagnostics. We can only rely on symptoms and signs such as fever, vomiting etc”* (FGD-6, Port health officer)

“*We lack quarantine facilities*. *Because if we don’t have these facilities*, *it means that we can create a space but there will be stigmatization”* (FGD-3, Custom officer).

Knowledge gain (level 2): Participants reported improved knowledge and skills in outbreak detection, surveillance, diagnosis, coordination and case management, along with a better understanding of their roles:

“*Our skills and knowledge in infection prevention and control, laboratory surveillance, and laboratory diagnostics, coordination have improved since the FSX”* (KI-4, Surveillance officer)

*“After the FSX*, *I now know my role in terms of pandemic prevention and response”* (FGD-5, Veterinary officer).

Skill application (level 3): Participants successfully integrated the FSX learnings into their working practices, improving responses to real-world outbreaks like COVID-19:

[…] “*our team at the border at the sub-county level, Kajiado central, did a lot because we were to implement all what we learned during the FSX”* (KI-4, Surveillance officer)

“*COVID-19 came some few months after the FSX*. *So we had a better idea of how to tackle COVID-19”* (KI-8, Veterinary officer).

Results (level 4): Participants reported that the FSX had a positive impact on infectious disease preparedness and response in Namanga and beyond:

“*The FSX helped in identifying gaps in our public health response plans and the standard of Procedures (SoPs). It helped build capacities around. The FSX help them to some extent in managing COVID-19”* (FGD-1, Immigration officer)

“*And this FSX has helped us in several ways*: *Firstly*, *in terms of coordination*, *secondly*, *teamwork and reaction time*. *We were really able to come to a point with the real cases and how to deal with them at the right time”* (KI-4, Surveillance officer).

### Limitations of the FSX

One of the themes that surfaced was the limitations of the exercise in terms of sustainable results.

A major challenge identified was frequent staff turnover. This was particularly problematic during the COVID-19 pandemic, as most trained personnel were no longer present. Participants expressed concerns about repeated training of the same individuals and their loss to other organizations (UN, GIZ etc):

*[…]* “*Most of my colleagues were not here during COVID-19. Because some of the people who took part in the FSX have been transferred to other places”* (FGD-8, Port health officer)*.*

“*Unfortunately or what I have seen from where I come from is that there are specific people who continually are trained and retrained and once they exist to GIZ*, *to FAO*, *to UN*, *Africa Union*. *And once they leave*, *we lose all the institutional knowledge they gained from the exercise”* (KI-2, Veterinary officer).

The participants reported that some lessons learned during the FSX were implemented afterwards, while others were not:

*“Few things like hand washing facilities were done but other things were not done after the FSX” (KI-2, Veterinary officer).*

The study identified resource competition as a potential barrier to the FSX effectiveness and sustainability:

“*We have many competing needs for the resources we already have to support the FSX” (KI-3*, *Epidemiologist)*.

### Cross-border coordination

Challenges in information sharing between Kenya and Tanzania also hinder efficient cross-border response:

“*We have found that the exchange of information across the border is very limited. Given the lack of a coordinated system for information and data exchange, this could have contributed to faster cross-border information exchange and trust within the system. It would have also made us work effectively”* (KI-14, Environmental Health Officer).

### Sustainability

Participants noted that stakeholder funding and commitment is critical to the sustainability of the FSX, and that a joint effort between the EAC and the border districts could ensure long-term success:

“*Maintaining and keeping the system working throughout requires a lot of effort on both member states that are working together in the East Africa Community […] but there is a need to call up all the border stakeholders or the counties within the borders*. *Which can effectively make these activities to be sustainable”* (KI-4, Surveillance officer).

## Discussion

This study addresses a gap in the literature by presenting empirical data on the effectiveness of FSX in enhancing cross-border collaboration and response to infectious diseases. The study explores the qualitative aspects of preparedness and response by including a FGD and KI interviews. This complements the existing literature’s focus on quantitative measures. Although the findings are context-specific, they may be relevant to comparable border regions. The FSX exercise prior to the COVID-19 outbreak provides valuable insights into the practical impact of the exercise during a real-life event.

Despite the limitations of the FSX, participants were satisfied with the FSX as it was instrumental in improving their preparedness and response capacity. The knowledge and skills gained during the FSX were directly applied to the response to COVID-19 and other infectious disease outbreaks, resulting in immediate improvements at the PoE. These included better case management, faster response times, development of SOPs and response plans, improved coordination and communication between sectors, etc.

This aligns with prior research on the value of SimExs [14, 15, 22]. The COVID-19 pandemic further highlighted the need for skills and capacity for cross-border disease prevention and response [8, 22, 23]. However, this study suggests that FSXs, when implemented alone and not as a coherent training programme, may overlook ongoing knowledge transfer and systemic issues, thereby compromising their overall effectiveness, impact and sustainability in improving responses to infectious disease outbreaks.

The challenges of planning, logistics, and sustainability are crucial to the effectiveness of emergency response efforts, as demonstrated by COVID-19 [24]. A successful pandemic response requires precise cross-border coordination among various agencies, resources, and priorities, often leading to significant logistical complexities [24, 25]. This study highlights that sustaining these efforts over time and implementing post-exercise activities are particularly challenging due to the need for consistent funding. For long-term sustainability, a dedicated budget from national and regional governments, supported by vertical programs, is essential to ensure coordinated efforts and minimize duplication [26].

It was possible to identify effects of the Namanga FSX corresponding to all levels of Kirkpatrick’s model, although for attribution at level 4 it would have been necessary to have objective pre- and post-exercise assessments rather than just self-reported evaluation.

SPAR scores within the IHR MEF for both countries improved in several key areas after the FSX, particularly in capacities C5 (surveillance), C9 (infection prevention and control), and C11 (PoE and port health). These gains, reflected in 2019 SPAR scores and sustained through 2022, highlight the sustainable impact of the FSX on health security in both countries. However, further improvements are needed in capacity C11, specifically in Indicators C11.1 (core capacity requirements at airports, ports, and ground crossings) and C11.2 (effective public health response at PoE) [27].

This study demonstrates the limitations and challenges in fully realising the benefits of an FSX, emphasizing the need for long-term financial planning and dedicated budgets for post-exercise activities. Staff retention issues and rapid turnover, for example where trained participants leave for better employment opportunities in bilateral and multilateral organizations, weakens the sustainability of the effect of interventions like FSX.

However, the retrospective nature of the study has its limitations, as participants may find it difficult to recall specific FSX details, and the data also tend to support a hypothesis that those who were motivated to respond to the follow-up survey, were those who most felt they had benefited. The inclusion of various FSX stakeholders, including facilitators, enriched the findings but may have introduced response bias, potentially influencing the results. The analysis of the effectiveness of the FSX would have benefited from a pre-exercise survey which tested knowledge, not just perception, and this would be a clear recommendation for future such exercises. Future research could explore cost-effectiveness analysis and knowledge transferability, including when trained individuals move into new organisations and share expertise with colleagues who were not part of the training. To minimise potential study limitations, a diverse sample of participants from both countries and different roles within the FSX was ensured to capture different perspectives; an anonymous survey was used to reduce response bias, quantitative data were cross-referenced with qualitative findings to validate findings. The survey instruments, the KII interview guide and the FGD guide were piloted prior to data collection.

### Recommendations

1. **General Recommendations:**
  - **Earlier planning:** Conduct FSXs with advanced preparation, focusing on effective coordination and communication.
  - **Comprehensive training:** Extend training duration, include diverse infectious diseases, and cover collaboration, negotiation, and information disseminations skills.
  - **Pre-training:** Incorporate orientation sessions, rehearsal, and smaller-scale drills before the main FSX. Ensure that participants are well-versed in preparedness tools in their own environment and have clearly defined roles.
  - **Regular FSXs:** Establish well-coordinated structures to conduct regular FSXs based on disease prevalence to maintain participants’ preparedness, skills, and knowledge, while keeping them updated on emerging and re-emerging diseases.
2. **Recommendations for transboundary capacity in Kenya-Tanzania:**
  - **Address resource limitations:** Equip Kenyan and Tanzanian One Stop Border Posts and the Namanga Border Health Centre with dedicated quarantine areas, transport, PPE, and diagnostic laboratories.
  - **Enhance logistics and networks:** Improve the logistical arrangements and address the network challenges during the next FSX.
  - **Strengthen information sharing:** Establish a coordinated cross-border information sharing system between Kenya and Tanzania.
  - **Sustainability:** Secure post-FSX funding through national/sub-national government collaboration to support follow-up activities and promote long-term sustainability of the FSX.

## Supporting information

S1 Fig Post FSX survey questionnaire. (PDF)

S2 Fig Interview guide. (PDF)

S3 Fig Focus group discussion guide. (PDF)
