# Supplementary material for: Evaluation of a cross-border field simulation exercise on the response to outbreaks of infectious diseases in Namanga, Kenya and Tanzania
Source: PLOS Glob Public Health. 2024 Oct 16;4(10):e0003832. doi: 10.1371/journal.pgph.0003832 (PMC11482668; doi:10.1371/journal.pgph.0003832)
Supplement: S1 Fig — (PDF) [file pgph.0003832.s001.pdf]

S1 Fig. Post FSX Survey Questionnaire.

Section 1: Demographics

1. What is your age range?
   o 18-30
   o 31-40
   o 41-50
   o 51-60
   o 61-70
   o 71 and above

2. What is your gender?
   o Female
   o Male
   o Other
   o I prefer not to answer

3. Please select your highest academic or professional degrees/certification
   o PhD/ DPH / Dr.
   o MSc, MA, MPH
   o BSc., BA
   o Diploma, high school
   o Other
   o I prefer not to answer

4. In which profession or discipline do you work?
   o Human medicine
   o Veterinary medicine
   o Health sciences
   o Social sciences
   o Environmental health

5. How long have you been working in this profession?
   o 0-2 years
   o 3-5 years
   o 6-10 years
   o 10-15 years
   o 15 years plus

Section 2: this section asks about your role(s), experience during the Namanga FSX

6. Please choose from below what your function was during the FSX.
   o Facilitator
   o Participant
   o Role player
   o Observer
   o Evaluator
   o Support staff

7. I have gained new skills and knowledge from the FSX.

o Yes, I fully agree
o Yes, I partly agree
o Neither do I agree nor disagree
o No, I partly disagree
o No, I strongly disagree

8. Do you think the FSX was able to help identify effectively the best practices, challenges and lessons learnt in preparedness and response to outbreaks in Kenya and Tanzania?
   o Yes
   o No

9. Overall, how satisfied were you with the FSX?
   o Very unsatisfied
   o Satisfied
   o Neither satisfied nor dissatisfied
   o Dissatisfied
   o Very dissatisfied

Section 3: this section asks about your experience after the Namaga FSX

10. Did taking part in the FSX…
    o Improved your understanding and knowledge on infectious disease outbreaks? A. Yes B. No
    o Improved your confidence level on managing infectious disease outbreaks? Yes B. No

11. On a scale from 1-5, how well did the skills and the knowledge gained from the FSX prepare you in conducting your work in COVID-19 response and other infectious diseases?
    o 1 (very low)
    o 2
    o 3 (average)
    o 4
    o 5
    o 6 (very high)
    o I do not know

Section 4: this section asks your skills/knowledge in strengthening emergency preparedness and response

12. Did the exercise allow you to test your response capacity?
    o Yes, I fully agree
    o Yes, I partly agree
    o Neither do I agree nor disagree
    o No, I partly disagree
    o No, I strongly disagree

13. Did the exercise improved your understanding of your role and function during an emergency response?
    o Yes, I fully agree

o Yes, I partly agree
o Neither do I agree nor disagree
o No, I partly disagree
o No, I strongly disagree

14. Did the exercise helped your organization to identify some of your strengths as well as some of the gaps in your understanding of response systems, plans and procedures?
o Yes, I fully agree
o Yes, I partly agree
o Neither do I agree nor disagree
o No, I partly disagree
o No, I strongly disagree

15. As a result of the exercise, my organization was better prepared for a health emergency.
o Yes, I fully agree
o Yes, I partly agree
o Neither do I agree nor disagree
o No, I partly disagree
o No, I strongly disagree

16. How can the next FSX be improved? ____

Section 5: Questions for facilitators only

17. Did taking part in the exercise prepare you to plan and conduct simulation exercises in the future?
o Yes, I strongly agree
o Yes, I agree
o Neither do I agree nor disagree
o No, I disagree
o No, I strongly disagree

18. After taking part in preparing and implementation of the FSX, how confident are you now in taking a similar role in the future?
o 1 (very low)
o 2
o 3 (average)
o 4
o 5
o 6 (very high)
o I do not know

19. After taking part in preparation and implementation of the FSX, have you been involved in planning and conducting another simulation exercise in your country? A. Yes B. No

If yes, how many exercise(s)?

1 simulation exercise
2 simulation exercises
3 and above simulation exercises

Section 6: Participation in a follow-up study

20. Are you interested in participating in a follow-up study which involves a one-to-one interview to better understand the impact of the simulation exercise?

☐ Yes, please provide your email address …

☐ No
