# Supplementary material for: Evaluation of a cross-border field simulation exercise on the response to outbreaks of infectious diseases in Namanga, Kenya and Tanzania
Source: PLOS Glob Public Health. 2024 Oct 16;4(10):e0003832. doi: 10.1371/journal.pgph.0003832 (PMC11482668; doi:10.1371/journal.pgph.0003832)
Supplement: S2 Fig — (PDF) [file pgph.0003832.s002.pdf]

S2 Fig. Key Informant Interview Guide

Introduction:

1. Background of the participant

a. Name

b. Organization

c. Position

d. Years in position

2. Role in emergency situations:

a. Describe your role in emergency preparedness and response.

FSX Experience

1. Participation in FSX:

a. Have you participated in the regional cross-border FSX in Namanga in June 2019?

b. Did your organisation/government participate in the planning and execution of the Namanga FSX?

2. FSX Overview:

a. Briefly describe the regional cross-border FSX, including the scenario, purpose and your role.

3. Essential skills and knowledge:

a. In your opinion, what are the most important skills and knowledge required for an effective infectious disease emergency response in your role? Why?

b. How can the skills developed during the FSX be maintained and applied to real life emergency responses?

Retention and transfer of skills/knowledge

1. Lessons learned:

a. Can you recall the lessons learned that were highlighted during and after the exercise?

b. Were you involved in any post-exercise activities within your organisation to address the lessons identified?

2. Impact of the FSX on COVID-19 response:

a. How did the FSX help you, your team and your organisation to be better prepared for the current COVID-19 pandemic and other infectious diseases?

b. Was there anything missing from the exercise that would have improved your response?

3. Future preparedness:

a. As an arm of government/organisation, how do you plan to conduct various exercises to prepare for public health emergencies? Does the Namanga Joint Border Committee have a public health emergency plan?

Post-FSX experience

1. Further training needs:

a. What kind of training do you think is necessary to improve your personal level of preparedness to respond to an infectious disease outbreak?

2. Relevance of emergency exercises:

a. How relevant do you consider emergency exercises in preparing you for your current role?

b. How has your institution/unit better prepared for a public health emergency since the FSX? Is there room for improvement?

3. Importance of FSX:

a. Do you think conducting FSX is important?

b. What types of exercises, such as drills and table tops, should be conducted?

4. Exercise outcomes:

a. How do the FSX outcomes help your organization in response planning?

5. Participant selection:

a. How are participants selected for the FSX?

Impact of FSX

1.Acquired skills and knowledge:

a. What useful/relevant skills and knowledge have learned or developed from participating in the FSX that have helped you in COVID-19 response?

2. Frequency of exercise:

a. How often do you feel the need to participate in emergency prepared exercises to improve and maintain your response preparedness? (e.g., workshops, seminars, tabletop exercises, FSX?

End of Interview.
