# Supplementary material for: Evaluation of a cross-border field simulation exercise on the response to outbreaks of infectious diseases in Namanga, Kenya and Tanzania
Source: PLOS Glob Public Health. 2024 Oct 16;4(10):e0003832. doi: 10.1371/journal.pgph.0003832 (PMC11482668; doi:10.1371/journal.pgph.0003832)
Supplement: S3 Fig — (PDF) [file pgph.0003832.s003.pdf]

S3 Fig. Focus Group Discussion Guide

Introduction

Details of participants:

- Name
- Organisation
- Position held
- Years in position

Role in emergency situations:

- Describe your role in emergency situations, including emergency preparedness and response.

FSX Experience

- Participation in Namanga FSX:
- Did your organisation/government actively participate in the planning and execution of the Namanga FSX?

Retention and transfer of skills/knowledge

Reflection on lessons learned:

- Recall lessons learned during and after the exercise.

Post-exercise activities:

- Participate in post-exercise activities to address lessons learned.
- How did the exercise contribute to preparedness for the current COVID-19 pandemic and other infectious diseases?
- Identify any missing elements in the exercise that could have improved response capabilities.
- Does the Namanga Joint Border Committee have a public health contingency plan?

Post-FSX experience (perception of preparedness response)

Further training needs:

- Identify training needs to improve personal emergency preparedness to respond to infectious disease outbreaks.

Relevance of emergency excecises:

- Evaluate the relevance of emergency exercises in preparing for current role.

Institutional preparedness:

- Describe how the institution/unit has improved its preparedness for public health emergencies since the FSX.
- Suggest improvements for public health emergency preparedness.

Importance and frequency of FSX:

- Discuss the importance of conducting FSX and recommend the frequency.

Types of exercises:

- Suggest the type of exercises that should be conducted.

Outcomes and response plans:

- Evaluate whether the results of the exercises contribute to the improvement of the organisation's response plans.

End of discussion
